# Iron homeostasis imbalance and ferroptosis in brain diseases

**DOI:** 10.1002/mco2.298

**Published:** 2023-06-26

**Authors:** Haining Long, Wangshu Zhu, Liming Wei, Jungong Zhao

**Affiliations:** ^1^ Department of Diagnostic and Interventional Radiology Shanghai Sixth People’s Hospital Afliated to Shanghai Jiao Tong University School of Medicine Shanghai China

**Keywords:** ferroptosis, iron chelators, iron homeostasis, neurodegenerative diseases, stroke

## Abstract

Brain iron homeostasis is maintained through the normal function of blood–brain barrier and iron regulation at the systemic and cellular levels, which is fundamental to normal brain function. Excess iron can catalyze the generation of free radicals through Fenton reactions due to its dual redox state, thus causing oxidative stress. Numerous evidence has indicated brain diseases, especially stroke and neurodegenerative diseases, are closely related to the mechanism of iron homeostasis imbalance in the brain. For one thing, brain diseases promote brain iron accumulation. For another, iron accumulation amplifies damage to the nervous system and exacerbates patients’ outcomes. In addition, iron accumulation triggers ferroptosis, a newly discovered iron‐dependent type of programmed cell death, which is closely related to neurodegeneration and has received wide attention in recent years. In this context, we outline the mechanism of a normal brain iron metabolism and focus on the current mechanism of the iron homeostasis imbalance in stroke, Alzheimer's disease, and Parkinson's disease. Meanwhile, we also discuss the mechanism of ferroptosis and simultaneously enumerate the newly discovered drugs for iron chelators and ferroptosis inhibitors.

## Graphical abstract

As neurological diseases that seriously affect the survival and prognosis of patients, stroke and neurodegenerative diseases cause excessive accumulation of brain iron by disturbing the normal mechanism of brain iron absorption and export, accompanied by the outbreak of oxidative stress, which leads to programmed cell death called ferroptosis, and eventually leads to cognitive and motor dysfunction of patients.

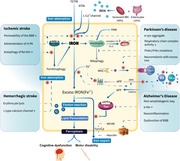

## 1 INTRODUCTION

Iron is a redox‐active element of life. Iron can accept or donate electrons to facilitate other redox reactions in the conversion between ferric (Fe^3+^) and ferrous (Fe^2+^) forms. Due to its redox ability, iron is widely utilized in nearly every biochemical pathway in the form of iron–sulfur (Fe–S) clusters and heme, ranging from oxygen transport as key a component for hemoglobin synthesis, to DNA metabolism as a cofactor for multiple enzymes including DNA repair enzymes and ribonucleotide reductase, to oxidative phosphorylation being an indispensable substance for complex I–IV of the mitochondrial respiratory chain, to myelin and neurotransmitter synthesis (e.g., as a cofactor for tyrosine hydroxylase).^1^, ^2^, ^3^, ^4^, ^5^ Therefore, balanced iron metabolism plays a critical role in maintaining the body's metabolism normal neurological function.

Iron accumulation in the brain has been reported to be closely related to a variety of neurological conditions in middle‐aged and elderly people, such as stroke, Alzheimer's disease (AD), and Parkinson's disease (PD). Local or extensive brain iron accumulation was found to affect the progression of these diseases, impairing the cognitive and motor ability of patients.^6^ In addition, iron accumulation triggers ferroptosis, which is a newly discovered iron‐dependent regulated cell death and different from autophagy, apoptosis, necroptosis, and other forms of cell death. The most essential biochemical attributes of ferroptosis are iron overload and the lethal accumulation of ROS and lipid peroxides accompanied by glutathione (GSH) depletion, glutathione peroxidase (GPX) 4 inactivation within the cell, leading to fatal damage and disorganization of the cell membrane at last.^7^ As a consequence of iron accumulation, ferroptosis is an important cause of neurodegeneration in brain diseases.^8^ Thus, elucidating how the central nervous system incorporates and manages iron under physiological and pathological conditions and how iron homeostasis imbalance cause ferroptosis is helpful to provide potential targets for the treatment of stroke and neurodegenerative diseases and provide clues to solve the puzzles in the field of imbalance of brain iron homeostasis.

In this review, we focus on (i) the mechanism of normal iron regulation in the brain involves iron absorption, storage, and export; (ii) iron accumulation in brain diseases, mostly focusing on stroke, AD, and PD; (iii) the mechanism of ferroptosis; (iv) the latest therapeutic agents for treating iron accumulation and ferroptosis, including iron chelators and ferroptosis inhibitors.

## 2 IRON HOMEOSTASIS IN THE BRAIN

### 2.1 Brain iron absorption

Most (90%) of the daily iron consumed by a healthy adult derives from the recycling of iron within senescent erythrocytes by reticuloendothelial macrophages in the spleen and liver, with the remaining 10% from dietary iron absorption by enterocytes.^9^ Iron recycled from erythrocytes and absorbed from diet enters the circulation as Fe^3+^.^10^ Fe^3+^ is more stable compared with Fe^2+^, which can generate ROS via the Fenton reaction, a type of reaction between Fe^2+^ and hydrogen peroxide (H_2_O_2_), with the ability to oxidize a wide range of organic substrates.^11^ Fe^3+^ that has lost three electrons cannot participate in the oxidation reaction again, making the transportation of Fe^3+^ in circulation relatively safe.

**FIGURE 1:**
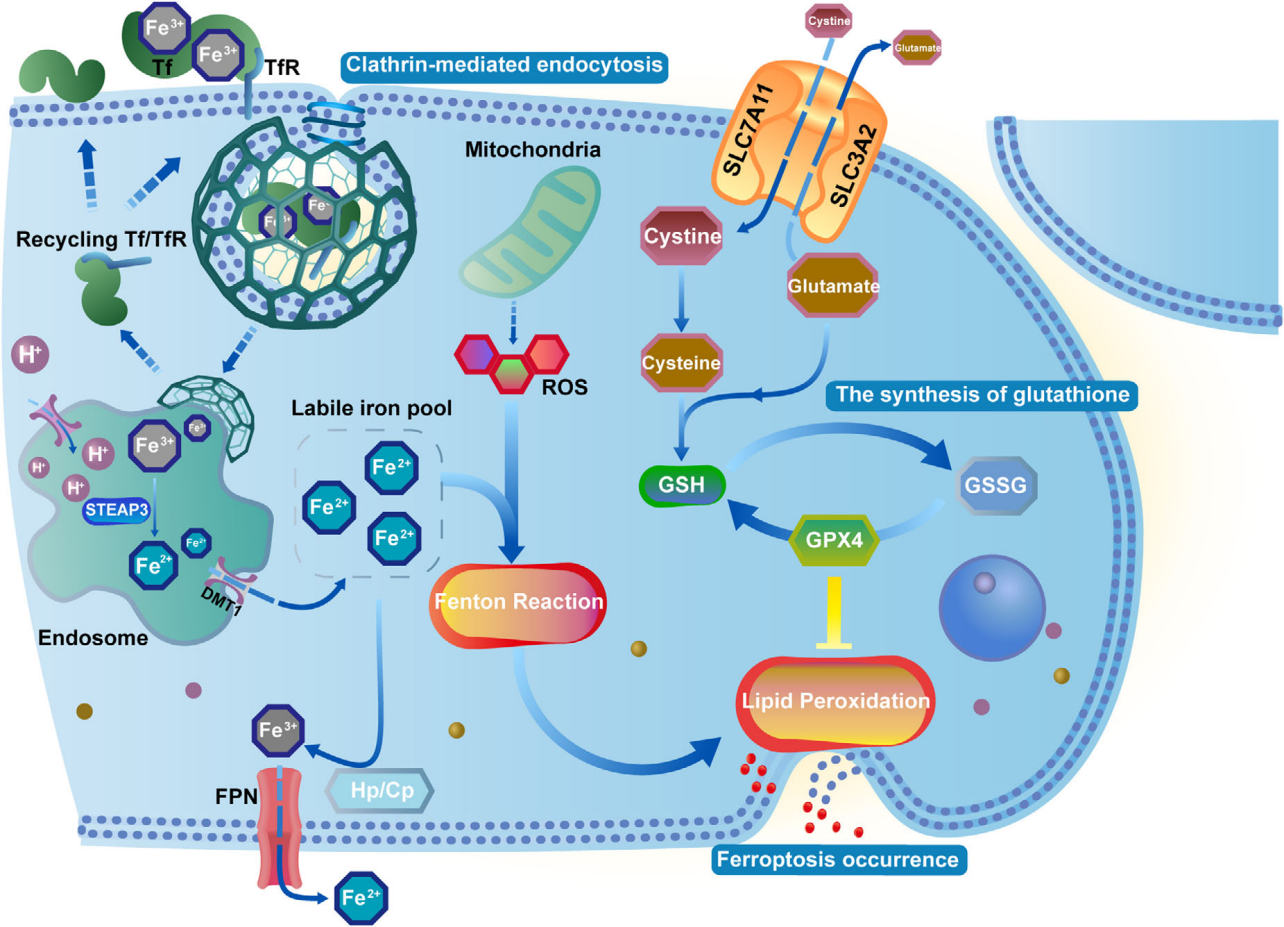
Metabolic processes of iron transport by transcellular pathway (endocytosis), lipid peroxidation related to the occurrence of ferroptosis, and the body's representative antioxidant system. The Fenton reaction stimulated by liable iron induces lipid peroxidation, while intracellular GSH is insufficient to resist it due to the rapid rise of liable iron so ferroptosis occurs due to the rupture of the unstable membrane. Tf, transferrin; TfR, transferrin receptor; DMT1, divalent metal transporter 1; FPN, ferroportin; Cp, ceruloplasmin; Hp, hephaestin; ROS, reactive oxygen species; SLC7A11, solute carrier family 7 member 11; SLC3A2, solute carrier family 3 member 2; GSH, glutathione; GSSG, oxidized glutathione; GPX4, glutathione peroxidase 4.

Iron is transported to the brain mainly in two forms: the iron–transferrin complex (TF–Fe^3+^) and the nontransferrin‐bound iron (NTBI). The regulatory mechanism of iron absorption in the brain is highly strict. The blood–brain barrier (BBB) is the first barrier for iron to enter the brain cells. The BBB consists of endothelial cells, astrocytic endfeet, pericytes, and basement membranes that collaborate to control the entry of substances or cells in circulation into the brain to maintain a stable environment for normal brain function.^12^, ^13^ And brain microvascular endothelial cells (BMVECs) are the main components of the BBB and also the target cells recognized by TF–Fe^3+^ complex.^14^ The uptake and recycling of TF–Fe^3+^ complex include several steps (Figure 1): (i) due to the tight junctions (TJs) between BMVECs, TF–Fe^3+^ complex can enter cells only in the transcellular pathway (endocytosis) by binding to transferrin receptor 1 (TfR1) on the membrane of BMVECs, which is clathrin dependent^15^; (ii) the internalized Tf/TfR1 complex is coated with membranes to form vesicles that are transported to the endosome and fuse with it; (iii) Fe^3+^ is released from Tf inside the endosomal compartment of BMVECs in an acidic pH (around 5.5) achieved by the activity of proton pumps; (iv) the reduction of Fe^3+^ to Fe^2+^ in the presence of ferric reductases, such as STEAP3^16^; (v) Fe^2+^ then crosses the endosomal membrane into the labile iron pool in the cytoplasm via divalent metal transporter 1 (DMT1)^17^, ^18^; (vi) return of non‐iron‐bound Tf (apo‐Tf) with TfR1 to the membrane where apo‐Tf is released into the blood.^19^ The vasculature of the brain is almost ensheathed by astrocytic end‐feet, which are specialized units that serve the function to maintain the ionic and osmotic homeostasis of the brain.^20^, ^21^ Once iron is released from the BMVECs, it may be taken up by the end‐feet of astrocytes. As the most abundant glial cells in the brain, the primary role of astrocytes is to transport iron ions to other glial cells and neurons rather than to store them.^22^ Before being expelled out of cells, Fe^2+^ is oxidized to Fe^3+^ by multicopper ferroxidases, including ceruloplasmin (Cp) and hephaestin (Heph). The major type of ferroxidase in astrocytes is Cp, while Heph is found in neurons, oligodendrocytes, and microglia.^23^, ^24^, ^25^, ^26^ The ferroxidase activity of Cp can facilitate iron export coupled to ferroportin 1 (Fpn1). After entering into interstitial fluid or cerebrospinal fluid in ventricles, Fe^3+^ binds with extracellular transferrin (apo‐Tf) readily and supplies iron to the cells expressing TfR1 within the central nervous system.^15^, ^27^, ^28^

NTBI is potentially toxic in the plasma, so it may still exist but can be barely detectable under physiological conditions.^29^ One transferrin molecule can bind 2 atoms of iron, and normally, there are left with ∼70% of the iron binding sites in the plasma transferrin pool are unoccupied at any time, which means transferrin saturation≈30%, thereby providing a considerable buffering capacity against the generation of NTBI.^11^ But TFs are over‐saturated under conditions of iron overload, resulting in NTBI redundancy. Due to its high redox activity, NTBI can react with biomolecules such as sugars, lipids, and proteins to cause peroxidative tissue damage by producing harmful ROS in cells.^30^ NTBI transport relies on DMT1 and ferric reductase in the traditional model. Fe^2+^, from the reduction of Fe^3+^ mediated by reductases, cotranslocates with H^+^ across the cell membrane, which is mediated by DMT1.^31^ Moreover, the translocation of NTBI by DMT1 is pH dependent. The increase of H^+^ in the local brain promotes the transport of NTBI, leading to the accumulation of Fe^2+^ in the impaired nervous system. Indeed, astrocytes, oligodendrocytes, and microglia take up iron in the form of NTBI in a certain proportion even under physiological conditions, given that their low expression of TF cannot satisfy physiological needs. However, NTBI uptake will be proportionately increased especially in cases of iron overload, which might aggravate intracellular oxidative stress.^32^ A recent study reports that ferritin (FT) heavy chain as a transport protein helps NTBI to cross the BBB by binding with T‐cell immunoglobulin and mucin domain‐containing 1 receptor 1 in human endothelial cells.^33^ In addition, prion protein, a ubiquitously expressed plasma membrane glycoprotein that is most abundant in neuronal cells, can promote the transport of NTBI mediated by DMT1.^17^

### 2.2 Brain iron storage and export

FTs, which serve as the major cellular iron storage protein, are both nontoxic and bioavailable forms in the sequestration of free iron (Fe^2+^).^34^, ^35^ Excess iron is mainly stored in the cytoplasmic FT or partly exported to the extracellular fluid to keep the intracellular labile iron pool relatively stable. In addition, as the major intracellular iron‐utilizing organelles where the Fe–S cluster is synthesized to keep the normal function of the respiratory chain complexes I–IV and provide a steady energy output, mitochondria also have their exclusive FTs, called mitochondrial FT (FtMt), to prevent the detrimental effect of oxidative damage, such as the release of cytochrome *c* and the inhibition of Fe–S cluster enzymes.^36^, ^37^, ^38^ Iron over the reserve is transferred out of the cell, which relies on Fpn1, the only‐known iron export protein on the cell membrane.^39^

Because of the essential function of Fpn1 in cellular and systemic iron homeostasis, the expression and activity of Fpn1 are regulated at multiple levels. At the cellular level, Fpn1 is regulated by the iron regulatory protein (IRP)/iron responsive element (IRE) system that maintains cellular iron homeostasis by regulating iron transport and storage.^40^ It controls the translation of proteins responsible for iron uptake (DMT1 and TfR1), storage (FT), and export (Fpn1). Under cellular iron deficiency, IRPs bind to the IRE, a stem‐loop structure in the 5′‐untranslated region of Fpn1 messenger RNA, to inhibit the translation of Fpn1, thereby limiting iron export.^41^ Furthermore, the IRP/IRE system can downregulate FT assembly and translation, and upregulate the expression of TfR1 and DMT1, so finally enhancing the absorption and reducing the storage and export of iron.^42^, ^43^, ^44^, ^45^ Under iron redundancy, the IRP/IRE system downregulates the translation of TfR1 and upregulates the translation of FT and Fpn1, so limiting the absorption while promoting the storage and export of iron.^46^ At the systemic level, Fpn1 activity is regulated by hepcidin, a 25 amino‐acid peptide hormone mainly expressed by the liver.^47^ The expression of hepcidin is influenced by several physiological conditions, such as systemic iron levels, inflammation, hypoxia, anemia, erythropoiesis, and infection. By binding Fpn1 on the cytoplasmic membrane, hepcidin is able to induce the ubiquitination of Fpn1 mediated by the E3 ubiquitin ligase RNF217.^48^ Subsequently, nonfunctional Fpn1 is under internalization and degradation, thereby inhibiting iron export to circulation.^49^ Moreover, it is reported that hepcidin functions by downregulating TfR1 and DMT1 in BMVECs, astrocytes, and neurons, which suggests that hepcidin can regulate iron import and export in the brain simultaneously.^50^, ^51^, ^52^, ^53^

Although the mechanisms by which the IRP/IRE system and hepcidin function differ, they reach the same goal with mutual dependence, and both are affected by intracellular iron levels.^45^ IRP1 and IRP2, which are the two forms of IRP protein, are the main regulators of cellular iron in humans. The regulation of Fpn1 by hepcidin is mainly dependent on IRP2. Animal studies have shown that IRP2 dominates the mouse brain, and IRP2 knockdown significantly affected the downregulation of Fpn1 regulated by hepcidin.^54^ Therefore, the regulatory pathway of hepcidin and IRP/IRE system partially overlap.

From what has been discussed above, we found that iron transport is chemically modified by a variety of enzymes and combined with different proteins. Through this sophisticated transport mode, tissues are effectively protected from iron‐mediated oxidative damage. With the normal function of BBB, the regulation of the IRP/IRE system, and hepcidin, brain iron metabolism can reach an equilibrium state. And it was reported to be largely unaffected even when peripheral iron homeostasis was dysregulated.^55^

## 3 IRON METABOLISM IN BRAIN DISEASES

Under physiological conditions, brain iron content is in a dynamic balance of uptake, storage, and exportation to maintain the normal metabolism and function of neurocytes. Brain iron homeostasis largely depends on the integrity of the BBB and the accurate regulation of related proteins at the systemic and cellular levels, including TF, TfR1, DMT1, FT, Fpn1, and so on.

### 3.1 Iron metabolism in stroke

Stroke is the second leading cause of death and disability in the adult population in modern society, including ischemic stroke (IS) caused by the obstruction of the artery, accounting for about 87% of all stroke cases, and hemorrhagic stroke (HS) mainly caused by arterial rupture, accounting for about 13%.^56^ The causal association between stroke and brain iron accumulation has long been confirmed by a large number of clinical data, including neuroimaging and basic medical research.^57^, ^58^, ^59^, ^60^ As the gatekeeper of substance homeostasis in the brain, BBB plays an important role in controlling the import of brain iron.^60^ Once the BBB is breached, the homeostasis of iron in the brain will be disrupted, along with the altered expression of iron‐related proteins. Though both IS and HS can trigger brain iron homeostasis imbalance, the pathways differ between the two types of strokes. Clarifying the differences between the two will be favorable for precision treatment in the future.

#### 3.1.1 Ischemic stroke

BBB has extremely low paracellular permeability and high electrical resistance due to the existence of TJs between adjacent BMVECs.^61^, ^62^ In IS, the paracellular permeability of the BBB increases greatly for the disruption of TJs, so free iron ions and FT in the circulation penetrate the brain parenchyma through weak barriers, resulting in iron overload in the brain. Recombinant tissue plasminogen activator (rt‐PA) is the preferred treatment for acute IS within 4.5 h of the onset of symptoms and has been proven effective in thrombolysis and recanalization of blood vessels.^63^, ^64^ However, the administration of rt‐PA increases the extent of BBB leakage, which may result in hemorrhagic transformation related to high mortality.^65^ Therefore, patients treated with rt‐PA are prone to severe iron overload, which will further aggravate neurological dysfunction caused by ferroptosis if combined with hemorrhagic transformation, even the risk of death. In addition, influenced by ischemia/excitotoxicity, the endocytosis of the Tf–TfR1 complex and expression of DMT1 in the ipsilateral ischemic region is increased without changing neuronal TfR1 levels, implying the circulation rate of TfR1 increased rapidly in response to iron overload.^66^, ^67^ Mass Tf–TfR1 complexes containing iron enter the endosome, releasing Fe^2+^ under an acidic medium, which finally overflows the labile iron pool.

A selective autophagy pathway known as ferritinophagy, a process in which iron‐loaded FT is transmitted to the lysosome for degradation along with the free iron release, occupies a critical place in systemic iron homeostasis.^34^, ^68^ The concentration of labile iron increased significantly along with reduced content of FT 6 h after middle cerebral artery occlusion (MCAO), suggesting that the course of ferritinophagy in cortical neurons is highly activated by stroke.^69^ In addition, Fe^2+^ can be released even after bounding to FT, which will be potentiated especially when the intracellular fluid gets acidic and in the presence of superoxide radicals.^70^, ^71^

The limited iron excretion is related to Fpn1 dysfunction. Recently, evidence shows that the microtubule‐associated protein tau, which regulates the stability and dynamics of microtubules in neurons, is closely related to the function of Fpn1.^72^ In addition to being the major component of neurofibrillary tangles in AD and frontotemporal dementia, tau is prone to progressive hyperphosphorylation in neural disease, which makes it easy to aggregate/deposit and interferes with normal cellular functions.^73^ Therefore, tau seems to contribute to the development of neurodegeneration. Patients were found to face more severe neurological damage following MCAO, including dilated motor function deficit, high frequency of epileptiform discharge activity, and markedly larger cerebral infarction, in the presence of tau.^74^, ^75^ The existence of tau connects iron overload with ipsilateral hemispheric stroke to some extent.^76^ Tau facilitates iron export by trafficking amyloid precursor protein possessed with ferroxidase activity to stabilize Fpn1, while this process is suppressed by ischemia‐reperfusion injury, resulting in intracellular iron retention as a new mechanism of iron accumulation after stroke.^76^, ^77^ Indeed, compared with wild‐type neurons, the iron export rate and half‐maximal lethal dose for Fe^2+^ toxicity were significantly lower in tau‐knockout neurons, which implies getting rid of excessive iron is anticytotoxic.^78^

Compared with iron accumulation, poststroke iron deficiency in the brain has rarely been reported. However, in recent years, there has been evidence linking IS to serum FT deficiency, or called anemia in stroke recovery.^79^ Anemia after IS was found to be associated with lower muscle strength and cognitive impairment in patients, so early identification of poor iron status might help to identify patients with poor functional outcomes after rehabilitation.^80^ However, the pathomechanism linking serum iron deficiency to IS remains unknown. Moreover, brain iron homeostasis is not vulnerable to circulating iron omeostasis imbalance due to the protection of BBB, so it remains to be unclear whether brain experiences a state of iron deficiency under the condition of serum iron deficiency after AIS and whether serum iron deficiency is associated with brain iron accumulation after stroke. Addressing these issues will provide new insights into improving iron chelation therapy.

#### 3.1.2 Hemorrhagic stroke

The excess iron in HS comes mainly from the intracerebral. The most common cause of HS is aneurysm rupture caused by hypertension. Mass blood‐borne cells, chemicals, and fluid from blood vessels invade local brain parenchyma across the ruptured vessel walls.^81^ Hematomas composed of numerous red blood cells are formed surrounded by the brain parenchyma. During the absorption of the hematoma, the red blood cells disintegrate and shed hemoglobin. The phagocytosis of erythrocytes by microglia is mediated by CD36, a well‐recognized integral cell membrane protein.^82^ On the one hand, microglia are activated to express mass heme oxygenases (HOs), which engulfs and decomposes heme within hemoglobin, on the other hand, they highly express FT to chelate iron.^83^, ^84^ Excess iron that cannot be chelated will either be transferred into neurons via the Tf–TfR1 system or released in the cytoplasm of microglia, both of which exert neurotoxicity.^85^, ^86^

In addition, It is reported that voltage‐gated calcium channels act as a facile conduit for the entry of free ferrous iron into neurons under iron overload conditions.^87^ L‐type calcium channel antagonists represented by nimodipine are used in the treatment of secondary arterial vasospasm after aneurysmal subarachnoid hemorrhage and intracerebral hemorrhage. Studies found that nimodipine may serve as a protective agent for cells highly susceptible to iron toxicity but with limited neurological function improvement.^88^, ^89^ Therefore, L‐type calcium channels seem to aggravate iron‐induced neurotoxicity, however, the fraction of free iron being imported through this pathway under iron overload conditions has not been elucidated. Whether the inhibition of this channel can achieve the desired effect of iron clearance without affecting ion transport in other cells (e.g., cardiomyocytes) is still unknown. Further studies are still needed to confirm the protective role of L‐type calcium channel inhibitors in preventing neurons from iron‐induced neurotoxicity after HS.

#### 3.1.3 Neurogliocytes in stroke

Neurogliocytes are the major regulators of the peri‐infarct/hematoma environment in the central nervous system and have been implicated in poststroke immune response and iron regulation.^90^, ^91^ The response of neurogliocytes in vivo would reflect the development of neuroinflammation after stroke to some extent. Therefore, tracking the response patterns of neurogliocytes could provide insight into finding targeted treatments for intralesional iron accumulation and neurological injury.

**FIGURE 2:**
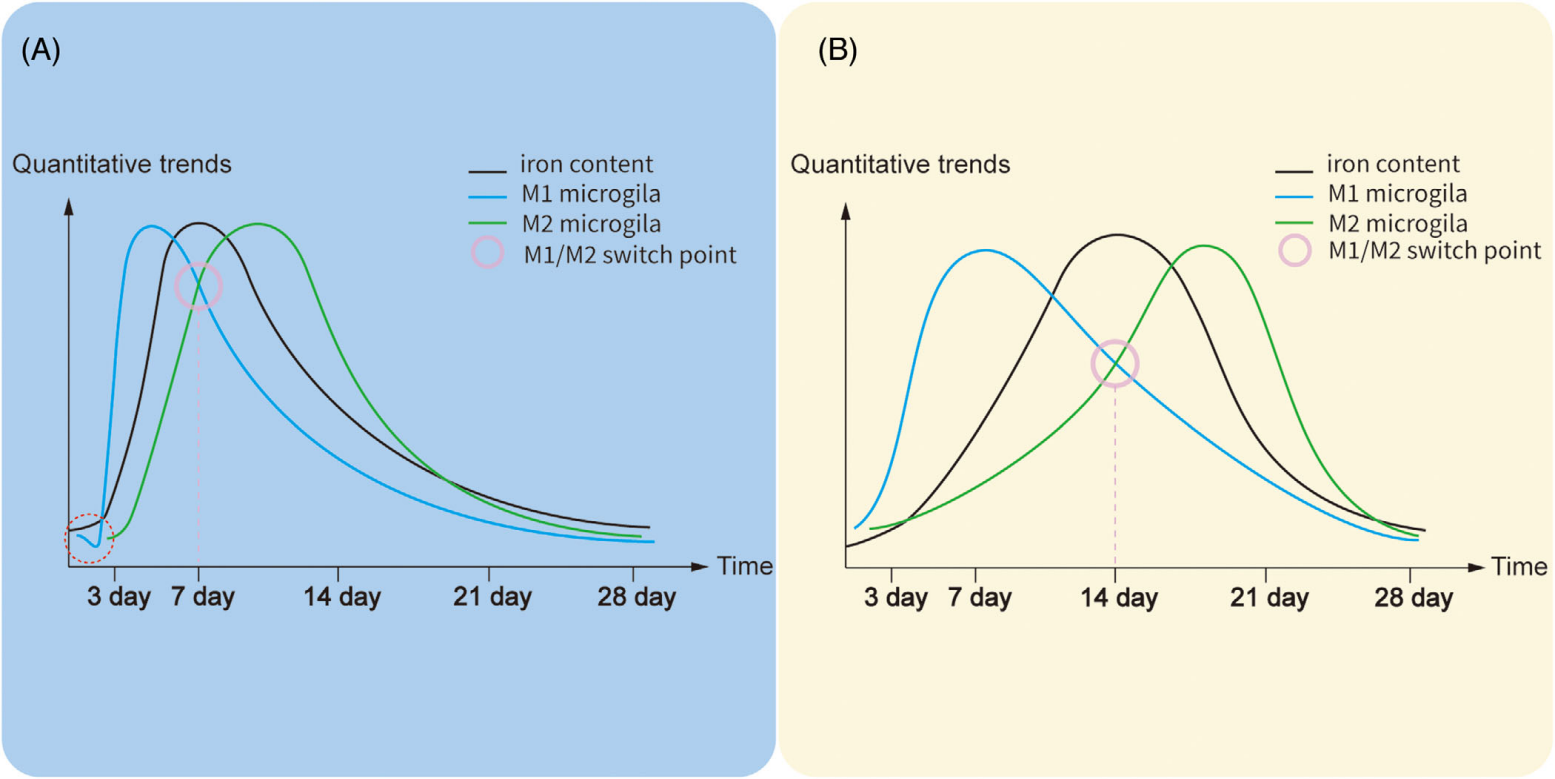
Timeline of microglia and iron after hemorrhage stroke (A) and ischemic stroke (B). These timelines are based on the data from the male rat model of AIS and HS.^95^, ^96^, ^97^, ^98^ Iron deposition in hemorrhage stroke is more rapid than in ischemic stroke due to the release of iron by the dissolution of mass red blood cells. The microglia numbers decrease (the red cycle) in the peri‐hematoma area shortly after HS, which might ascribe to necroptosis, and apoptosis caused by iron release from the hematoma, and significantly increase and peak at days 3−7. In contrast, microglia numbers in the core infarct area reach the peak during days 7−14 after IS and return to normal after day 28. Therefore, the reaction speed and proliferation ability of microglia cells were stronger in hemorrhage stroke, leading to an earlier peaking time of microglia proliferation as well as the earlier switch of the microglial phenotype (M1–M2).

Microglia, as the resident immune cells in the brain, play a fundamental role in mediating poststroke neuroinflammation and iron absorption, usually react faster than astrocytes to pathological stimuli, thereby regulating and activating nearby astrocytes and microglia.^92^ After stroke, microglia is capable to switch phenotype from proinflammatory (M1) to anti‐inflammatory (M2) phenotype so facilitating immune response to damage and repair in the nervous system, and the two phenotypes coexist but predominate at different stages.^93^ In murine models of IS, the M1 phenotype predominates during the first two weeks of injury, which corresponds to the upregulated expression of FT heavy chain and FT light chain, suggesting that the phagocytosis of microglia is highly active to iron in the early IS. In contrast, the M2 phenotype was found low at the early stage and gradually increases and predominates over two weeks, which corresponds to the increased main proteins in the myelin sheath, suggesting that the remyelination of damaged axons and neurologic repairment by using iron is under process in the later period.^94^ Compared with IS, the M1–M2 phenotype switch occurs earlier in HS (Figure 2).^95^, ^96^, ^97^, ^98^ Augmented M2 activation promotes vascularization in the injured zone, thus contributing to improvements in both tissue reorganization and functional recovery. A recent study shows that the interferon regulatory factor (IRF) 5–IRF4 regulatory axis functions to switch the polarization of microglia in stroke brains and change the outcome of stroke by tilting the axis.^99^

Astrocytes and oligodendrocytes play a crucial role in injury and repair response after stroke. Astrocytes are the most abundant glial cells in the brain, playing an important role in the blood–brain barrier maintenance, neuroinflammatory response, and the absorption and transport of iron.^29^, ^100^, ^101^, ^102^, ^103^, ^104^, ^105^ As a buffer pool of iron, astrocytes possess a high‐speed capacity for iron transport mainly due to their high expression of TfR1, DMT1, Fpn1, and Cp, which facilitates taking up iron from the abluminal side of BMECs and mobilizing it into the brain interstitial fluid, precisely regulating the iron concentration in neurons.^23^, ^29^ In response to stress, NADPH oxidase (NOX) 4 inside astrocytes was quickly activated to produce ROS, which further induces NF‐κB signaling and upregulates inflammation and lipocalin‐2 (LCN2) gene expression. LCN2 is capable to promote iron accumulation in neurons and glial cells by acting as an iron transporter,^106^, ^107^, ^108^, ^109^ and triggers the shift of astrocytes from an anti‐inflammatory towards a proinflammatory phenotype and contributes to BBB breakdown.^110^, ^111^ Therefore, astrocytic response serves an important role in oxidative stress and brain injury following stroke. Oligodendrocytes possess the highest iron levels in the brain due to their metabolic needs associated with the process of myelin formation, accurate wrapping, and repair in various brain diseases. Microglia and astrocytes are capable of releasing Fe^3+^ contained in FT to support oligodendrocytes for myelination or remyelination.^112^ However, as iron over accumulates after stroke, oxidative stress can damage oligodendrocytes and myelination, thus amplifying tissue damage.^113^ Compared with mature oligodendrocytes, oligodendrocyte progenitor cells (OPCs) are more susceptible to excitotoxic injury.^114^ Combined with recent research that hemoglobin causes damage to mitochondrial function in primary cultured OPCs, inhibits OPCs proliferation, and even cell death, it should be emphasized the importance of protecting OPCs against cell death after stroke to reduce brain injury and promote recovery.^115^, ^116^

Therefore, controlling the response of neurogliocytes, like the phenotype switch of microglia, the expression of inflammatory mediators like LCN2, and protecting OPCs might provide attractive therapeutic targets for stroke. However, it also should be noted that though M1 microglia might increase inflammation levels in the brain, the long‐term and improper inhibition of the M1 microglia might cause an adverse effect on the phagocytosis to iron and other fragments, thus leading to the deterioration of neurological function. Similarly, irrational or overused administration of iron chelators might interfere with the remyelination of damaged axons by oligodendrocytes during the recovery, thus causing poor outcomes though iron chelators have been proven effective in treating iron overload.

#### 3.1.4 Delayed iron deposition after stroke

Iron is an important component of the redox balance in the nervous system, and the regulation of brain iron homeostasis is of great significance to the recovery of neurological function after stroke. Despite breakthroughs in treatment of acute stroke in recent years, which have greatly improved the survival rate of stroke patients, how to reduce the long‐term functional impairment for stroke patients is still a matter of concern. The disruption of brain iron homeostasis after stroke will not only affect the ischemic areas but also impose a long‐lasting effect (the delayed iron deposition) on the remote but functionally or anatomically connected tissues, which get disconnected for primary ischemic injury. The delayed iron deposition would not be observed shortly after stroke but is gradually emerging over time. With a progressive increase in locally deposited iron, neurodegeneration of corresponding brain areas is obvious progressively, which eventually leads to poor outcomes for patients.^117^ As early as 1991, A Tamura et al.^118^ found that ipsilateral thalamic atrophy 1 year later, as well as alterations of the thalamic MRI signal, follows distant infarcts caused by MCAO, which is initial evidence for stroke‐related remote change. In 2017, Kuchcinski et al.^119^ found in a longitudinal study that thalamic R2* signal remains unchanged in the subacute phase of stroke (24–72 h after symptom onset) but was elevated 1 year after the cerebrovascular acute event and confirmed that infarcts in different regions of the brain lead to delayed iron deposition in the corresponding nuclei of the thalamus and ultimately worsen cognitive function in patients, which provides important evidence for the link between delayed iron deposition and neurodegeneration. In 2019, Linck et al.^120^ found infarcts of the striatum caused chronic iron deposition in the ipsilateral SN. Further, the asymmetry of bilateral iron deposition signal captured by MRI R2* is positively correlated with the poorer motor function of the nondominant arm. In other words, chronic iron deposition in the ipsilateral SN after stroke aggravates the defective motor ability of patients. Therefore, the evidence of neuroimaging above shows that stroke not only disrupts the brain iron homeostasis at the early stage of the disease but also changes the distribution of brain iron even after recovery, thus affecting the motor and cognitive functions of patients in the long term. Furthermore, histological studies demonstrate this association by verifying that the neurodegeneration of SN and thalamus induced by striatal lesions or corticothalamic disconnection respectively are related to the increased number of iron‐containing macrophages and/or microglia and extracellular iron deposition.^121^ The foundation of these collaborative changes lies in a large array of functional connections between different brain regions. This provides us with a new perspective for understanding the different patterns of iron deposition in the brain responding to the different stroke regions.

### 3.2 Iron metabolism in AD

Alzheimer's disease is a progressive and irreversible disease of neurodegeneration by far, which is typically characterized by an abnormal deposition of β‐amyloid (Aβ) plaques in extracellular plaques and neurofibrillary tangles formed by phosphorylated tau in neurons.^122^ Studies have found that iron content in the AD brain is significantly increased, which promotes the cognitive deficits of AD brains.^123^, ^124^, ^125^, ^126^ The association of iron accumulation with AD pathology is demonstrated in quantitative susceptibility mapping (QSM), which is highly sensitive to tissue iron, further confirming the reliability of iron as a biomarker for AD progression.^127^, ^128^, ^129^ Therefore, elucidating the mechanism of iron metabolism disorder in AD is crucial for its future treatment. Although the exact mechanisms of iron accumulation in AD remain unclear, it was thought to be involved in several following mechanisms: (i) Aβ plaques aggregation; (ii) phosphorylated tau deposition; (iii) neuroinflammation; (iv) dysfunction of BBB.

#### 3.2.1 Aβ and iron accumulation: a vicious cycle

Aβ generates from the amyloidogenic processing of Aβ precursor protein (APP), a ubiquitously expressed type 1 transmembrane protein in cells.^130^ The amyloidogenic way starts from the endocytic internalization of APP on the cell membrane. Internalized APP is trafficked to endosomes via the endocytic trafficking regulator ADP ribosylation factor 6 (ARF6), where APP is cleaved by β‐secretase (mostly BACE1 in the brain) and γ‐secretase, along with Aβ releasing.^131^, ^132^, ^133^ In contrast, the nonamyloidogenic processing of APP, as the predominant way, generates neurotrophic sAPPα with the help of α‐secretase cleavage at the cell membrane.

Previous studies suggested that APP promotes neuronal iron export by stabilizing Fpn1, the only iron export protein of cells.^134^, ^135^ This means that the cleaving of APP would decrease iron export, either in the amyloidogenic or nonamyloidogenic way. However, sAPPα, instead of APP, was found to work on iron export by stabilizing Fpn1 in the latest research, which further proves the amyloidogenic processing of APP is a harmful way to iron retention.^136^, ^137^, ^138^, ^139^ Aβ is able to activate microglia and promote the production of lactoferrin by binding to the NLR family pyrin domain containing 3. The interaction between the iron‐bound form of lactoferrin and APP diverted neuronal APP endocytosis from the clathrin‐dependent pathway (amyloidogenic process) to the nonamyloidogenic process mediated by ARF6, further aggravating iron retention.^140^ Therefore, rational inhibition of the amyloidogenic process appears to be a viable approach to treating AD. However, the functional abnormalities reduction mediated by BACEi inhibition in AD brains is found unstable either in animal models or clinical trials in humans.^141^, ^142^ This may attribute to differences in patient populations, mouse models, or methods used, and whether compensatory pathways for the amyloidogenic process exist remains to be explored.

The interaction between iron retention with Aβ plaque within neurons appears to form a vicious cycle that promotes cytotoxicity. First, Fe^2+^ and Fe^3+^ play key roles in Aβs folding and aggregation. Iron ions, especially Fe^3+^, can promote β‐sheet formations on the Aβs C‐terminal, which is favorable to reducing negative surface charges of Aβs.^143^ When electrostatic repulsion between the negatively charged domains of Aβs reduces, the Aβs aggregation becomes energetically favorable. Second, excess intracellular iron prevents IRP from binding to IREs in the 5′ untranslated regions of APP mRNA, leading to elevated APP generation, which could contribute to Aβ pathology partly.^144^ In addition, Fe^3+^ can be reduced to Fe^2+^ by binding plaque Aβ along with the generation of H_2_O_2_, thus propagating a continued source of ROS, and contributing to lipid peroxidation levels.^145^, ^146^, ^147^ This seems to explain how AD, as a progressive disease, promotes the production of its key pathogenic substances continuously by using the imbalance of iron homeostasis as a mediator, leading to a high redox state in the nervous system, neuronal vulnerability to ROS, and eventually develops into a neurodegenerative disease.

#### 3.2.2 Phosphorylated tau: decreased iron export

Tau, as the major component of neurofibrillary tangles, functions to mediate iron export from cells by trafficking sAPP to stabilize Fpn1.^122^ Loss of soluble tau protein due to deposition in neurofibrillary tangles may lead to iron retention. It was reported that the sites of iron deposition were found spatially colocalized with areas of tau burden, which further confirms the association between iron homeostasis disorder and tau pathology.^148^

As described in AD, Aβ deposition can cause iron homeostatic imbalance in the AD brain. The formation of neurofibrillary tangles follows after that of Aβ plaque in AD. Iron accumulation is able to enhance the hyperphosphorylation and aggregation of tau by stimulating the activity of the cyclin‐dependent kinase 5 complex and glycogen synthase kinase‐3β kinase.^149^, ^150^ In addition, HO‐1 protein can be detected in the neurofibrillary tangles in AD brains.^85^ On the one hand, elevated HO‐1 reduces the expression of memory‐related synaptic proteins, aggravates iron accumulation, and enhances tau phosphorylation in HO‐1 transgenic mice.^151^ On the other hand, Mass generation of HO‐1 in AD may lead to the metabolism of heme from damaged mitochondria, thus releasing Fe^2+^, and finally increasing oxidative stress level and neuronal damage.^152^ Slight cognitive impairment in AD patients has been shown to correlate with the increased concentration of HO‐1 in the frontal cortex and hippocampus.^153^

#### 3.2.3 Neuroinflammation

Neuroinflammation, resulting from microglia activation, is another hallmark of AD. On the one hand, the deposition of Aβs attracts chemotaxis of microglia and drives neuroinflammation by activating NLRP3 inflammasomes in microglia.^154^, ^155^ Meanwhile, NLRP3 inflammasome activation is also amplified by the interaction between Aβs and the adaptor protein apoptosis‐associated speck‐like protein containing a CARD (ASC), thus further recruiting and activating procaspase‐1 and contributing to pyroptotic neuron death.^156^ On the other hand, disruption in iron homeostasis triggers microglial polarization to the proinflammatory (M1) phenotype via ROS, resulting in a higher secretion of proinflammatory cytokines in microglia, such as TNF‐α and IL‐1β, and further aggravating neuroinflammation.^157^ And IL‐1β and TNF‐α function to promote iron retention in neurons by upregulating TfR1 and DMT1 and downregulating Fpn1.^158^ In addition, despite a strong resistance to iron accumulation of reactive astrocytes, astrocytes can still be activated by Aβs in AD, then releasing proinflammatory mediators and cytotoxic molecules, leading to neuroinflammation, and AD pathology exacerbation.^22^, ^159^

#### 3.2.4 Dysfunction of BBB

The ε4 allele of the apolipoprotein E (APOE) gene is validated as the strongest genetic risk factor for late‐onset AD and greatly affects the progression of Aβ pathology.^160^ APOE‐ε4 can reduce the expression of aquaporin‐4 (AQP4), a set of perivascular water channels, contributing to aberrant Aβ plaque accumulation.^161^ Notably, the BBB dysfunction caused by APOE‐ε4 in the capillaries is similar to the pathomechanism of cerebral small vessel disease, where iron leakage occurs in the brain due to BBB dysfunction.^162^ APOE‐ε4 and mild cognitive impairment were associated with higher cortical iron in a neuroimaging study using a 7.0T QSM, which may suggest iron leakage by impaired BBB in an APOE‐ε4 carrier.^163^ However, the association between APOE gene and iron accumulation seems to be less than strong in iron heritability by APOE.^148^, ^164^ In addition, vascular permeability factors secreted by activated astrocytes, such as vascular endothelial growth factors, can participate in the dysfunction of BBB and may affect the association between APOE and iron status.^165^ Therefore, the relation between APOE genotype and brain iron status remains elusive and to be researched.

### 3.3 Iron metabolism and PD

Parkinson's disease is the second most common disorder after AD, with a global prevalence of more than 6 million individuals.^166^ The major pathological hallmarks of PD include (i) the progressive iron deposition along with loss of DA neurons in the substantia nigra pars compacta (SNpc); (ii) the misfolding and aggregated α‐synuclein (α‐syn) result in the formation of Lewy bodies.^167^ The correlations between iron deposition and loss of DA neurons in the SNpc of PD brains have been proven in neuropathological and imaging studies, indicating that the imbalance of iron homeostasis participates in the pathology of PD progression.^168^, ^169^

#### 3.3.1 α‐Synuclein and iron accumulation: mutual reinforcement

α‐Syn is a water‐soluble protein consisting of 140 amino acids and highly enriched in the presynaptic membrane.^170^ Although the physiological function of α‐syn is remain unclear yet, it may play a role in maintaining the synaptic function, regulating the storage and release of synaptic neurotransmitters.^171^ In PD, α‐syn goes from the physiological monomer form to the pathological oligomers, protofibrils form, and aggregates to insoluble clumps after stepwise modification. Finally, α‐syn aggregates and interact with membranous organelles to produce Lewy bodies.^172^ Postmortem analysis of patients with PD shows that iron and α‐syn coexist in the Lewy bodies in the SNpc, suggesting an interaction between iron deposition and α‐syn pathology.^173^ Indeed, α‐syn aggregation plays a vital role in disrupting iron homeostasis. Guo et al.^174^ have reported intranasal injection of exogenous α‐syn preformed fibrils can cause iron deposition in the substantia nigra–striatum system of monkeys. On the one hand, as an iron‐reductase and iron‐binding protein, α‐syn is able to reduce Fe^3+^ to Fe^2+^, increasing the intracellular ferrous iron content, thereby increasing the generation of ROS and oxidative toxicity.^175^ On the other hand, many studies show the expression of α‐syn is associated with the Tf–TfR1 pathway of iron uptake. It has been reported that α‐syn enhanced clathrin‐mediated endocytosis in neuronal cultured cells.^176^ The hidden mechanism of this interaction was uncovered by recent evidence that α‐syn accelerates the rate of synaptic vesicle endocytosis alongside reducing the fraction of released synaptic vesicles through its activity to enrich the plasma membrane with phosphatidylinositol 4,5‐bisphosphate and phosphatidylinositol 3,4‐bisphosphate.^177^ Additionally, decreasing TfR1 and increasing FT were found in α‐syn‐knockout cells in culture, indicating that α‐syn functions to promote iron import and inhibit its storage, which is consistent with previous research.^178^, ^179^ The research above further describes evidence for a pathogenic role for α‐syn in dysregulating iron in PD.

At the same time, a lot of research has studied the role of iron in α‐syn aggregation and the regulation of posttranslational modification of α‐syn. It evidenced that α‐syn oligomerization and iron accumulation is a mutually reinforcing process. On the one hand, iron promotes α‐syn aggregation by binding to α‐syn and inducing its conformational changes. On the other hand, increased iron grabs IRP from the α‐syn mRNA, thus promoting the translation of the α‐syn mRNA.^144^ However, the link between iron and α‐syn goes deeper than this. A recent study reported that iron enrichment occurring in particular areas of PD brains affected the spreading pathology of α‐syn in mice models.^180^ Striatal iron retention alters the connectivity of the brain connectome, leading to a redistribution of α‐syn aggregates in the brain, or rather the restriction of phosphorylated α‐syn spread. Previous research with PD patients has reinforced this conclusion, suggesting iron modulates the degree of cross‐talk between the striatum/substantia nigra and cerebral cortex.^181^, ^182^ In addition, iron levels in the brain are known to increase with age, which seems to provide a reasonable explanation for the recent research that task functional connectivity in brains of the elder was found less strongly correlated as compared with the younger.^183^ Therefore, the rise in brain iron levels seems to play a protective role in PD to a certain extent; however, whether the protective effect of brain iron is related to its content or the different stages of PD remains to be explored.

#### 3.3.2 Neuromelanin: storage of iron and spark of neuroinflammation

Another typical neuropathological feature of PD is the depigmentation of dopaminergic neurons containing neuromelanin (NM) in SNpc along with the iron deposition throughout PD.^184^ Neuromelanin is the main iron chelator in SN neurons due to the low expression of FT, which plays its role mainly in the glia.^185^ Similar to FT, neuromelanin also sequesters iron by binding it in a redox‐stabilized ferric form and forming iron oxide clusters.^184^, ^186^ Zecca et al.^187^ examined metal contents in neuromelanin and showed that per gram of neuromelanin from SN contained 10.9 μg iron, which confirms the ability of neuromelanin to chelate iron. As an excellent chelator of iron, neuromelanin protects neurons from toxic compounds under physiological conditions.

However, excessive accumulation of NM in the course of PD increases the vulnerability of dopaminergic neurons and induces their death to a certain extent. Intraneuronal neuromelanin was found to increase the content of major histocompatibility complex class I (MHC‐I) in neuromelanin‐containing neurons in SN.^188^ MHC‐I functions to bind antigenic peptides and present them on neuronal membranes so that cytotoxic CD8^+^ lymphocytes could target neurons inducing their death.^188^, ^189^ Furthermore, recent neuroimaging studies also confirm the neuronal loss in dopaminergic neuromelanin‐pigmented neurons of the SNpc by providing evidence that dopaminergic function in striatum and substantia nigra negatively relates to NM‐sensitive signal and lateral SN R2* (on behalf of iron content).^190^, ^191^ After neuron death and collapse, neuromelanin is released into the extraneuronal space and slowly phagocytosed by microglial cells. The degradation of neuromelanin can not only activate microglial cells leading to the release of inflammatory and toxic mediators including TNF‐α and IL‐6 but also discharge toxic compounds and excess iron ions.^192^ In addition, IL‐6, via its trans‐signaling pathway, induces changes in the neuronal iron transcriptome that promote ferrous iron uptake and decrease cellular iron export via the cellular iron sequestration response.^193^ The increased inflammatory factors and iron cause the generation of free hydroxyl radicals in the cytoplasm, exacerbating the peroxidation of lipids and aggregation of α‐syn, resulting in PD progression and neuronal loss.

With the rapid development of neuroimaging research in recent years, the traces of iron and neuromelanin in PD have been gradually uncovered. 7T‐MRI brings us clear images in comparison of substantia‐nigra and R2* signal intensity between PD patients and the normal, intuitively showing elevated iron deposition and decreased neuromelanin in PD patients.^194^ The temporal changes of PD progression show that PD begins with striatal dopaminergic dysfunction, followed by SNpc iron accumulation preceding SNpc neuromelanin loss and, finally, dopaminergic cell loss, which suggests the interdependent changes between dopaminergic neurons, neuromelanin, and iron; moreover, brain iron deposition is more likely a consequence rather than a primary driver of neurodegeneration.^168^ The ability to detect neuromelanin with NM‐MRI and iron changes with R2^∗^ or QSM in the SNpc is valuable in helping the early diagnosis and monitoring progression of PD. More importantly, a comprehensive set of imaging biomarkers derived from neuromelanin and iron content in SNpc for PD diagnosis gives a better result than the detection of either alone and potentially, could be used to diagnose PD clinically.^195^

#### 3.3.3 Mitochondrial dysfunction and defective mitophagy

Mitochondria are the main organelles in the cells that produce ATP. Therefore, the functional stability of mitochondria is essential for cell survival, especially in neurons. Because brain is the most energy‐intensive organ in the body and consumes 20% of body oxygen for energy production, which implies that any disturbances of mitochondria function might cause oxidative stress and even neuronal death, for instance, iron overload. Iron plays an important role in the mitochondria and is mainly used in three pathways: heme synthesis, Fe–S cluster generation, and iron storage in the FtMt. Multiple evidence indicates that oxidative stress induced by mitochondrial dysfunction mainly due to decreased activity of respiratory chain complex I is critical to PD pathogenesis.^196^, ^197^ Treatment with complex I inhibitor rotenone was able to reproduce features of the PD in animal models, including SN dopaminergic degeneration, Lewy bodies generation, and oxidative stress.^198^ Recent research reported intracellular neuromelanin increased mitochondrial oxidative stress and exacerbated mitochondrial bioenergetic deficits mediated by heterocyclic aromatic amines, a substance that causes dose‐dependent cell death specifically to dopaminergic neurons potentially through inhibition of specific complexes of the electron transport chain.^199^, ^200^ In addition, it is also suggested that the accumulation of α‐syn in PD inhibits mitochondrial function by affecting the level of mitochondrial sirtuin 3 protein, an NAD^+^‐dependent deacetylase that regulates the acetylation/deacetylation level of complex I.^201^ The sirtuin 3 in mitochondria has been proven to prevent dopaminergic neuron loss in a rodent model of PD.^202^ This indicates that dysfunction of complex I plays an important role in mediating PD pathology. In addition, complex II–IV are also inhibited to varying degrees in PD, suggesting impaired mitochondrial oxidative respiration in PD.^203^ And this lessened capacity of mitochondrial electron transport chain is able to elevate mitochondrial iron uptake and ROS production.^204^

Mitophagy is a fundamental mechanism that regulates mitochondrial quality by a selective clearance of dysfunctional mitochondria in dependence on lysosomes.^205^ In this way, neurons can protect themselves from ROS damage and maintain physiological activity. It has been known to us that defective mitophagy due to Pink1/Prkn mutations leads to iron accumulation during a neurodegenerative process, such as PD.^206^, ^207^, ^208^ Recently, subcortical brain iron deposition was found highly predictive of mitochondrial impairment in patients with PD in vivo, which suggests a synergistic role between iron accumulation and mitochondrial damage in neurodegenerative diseases.^209^ Impaired mitochondria and defective mitophagy may disrupt iron homeostasis, and altered iron metabolism may, in turn, cause mitochondrial damage, forming a detrimental vicious cycle. Until now, it is still unclear whether mitochondria damage and defective mitophagy are causes or consequences of iron accumulation, but there is no doubt that both of them can accelerate PD progress and concur with the pathology of age‐associated neurodegeneration.

### 3.4 Other neurological diseases associated with iron metabolism

Huntington's disease (HD) is a progressive neurodegenerative disorder induced by the expansion of a CAG repeat in the huntingtin gene on chromosome 4, which leads to mutant huntingtin protein misfolding in neurons and microglial cells.^210^ Characteristic iron accumulation in HD patients occurs mainly in the striatum and cerebral cortex. The change of iron homeostasis in the HD brain may be associated with the mutation HTT. On the one hand, It was demonstrated that mutation of HTT upregulates the protein related to iron import, such as IRP1, Tf, and TfR, leading to iron accumulation in the striatum and cortex of HD mice.^211^ On the other hand, HTT mutation can disrupt mitochondria energy metabolism including inhibition of mitochondrial respiratory complexes II–IV, mitochondrial trafficking abnormality, and the impairment of mitochondrial, thus leading to abnormal ATP generation and neuronal death.^212^, ^213^, ^214^, ^215^ In addition, previous studies have evidence that microglial can promote the progression of HD under the activation of iron accumulation, however, the related mechanism is unclear. It was recently reported that iron contributes to HD progression and neurodegeneration by activating the microglial enzyme indoleamine‐2,3‐dioxygenase in the kynurenine pathway to generate increased amounts of neurotoxic intermediates 3‐hydroxykynurenine and quinolinic acid.^216^ This further supports a causal relation between iron accumulation, microglial activation, and neurodegeneration in HD and provides a potential target for the treatment of HD in microglia‐related mechanisms.

In addition, some of the rare neurodegenerative diseases with iron homeostasis imbalance involve amyotrophic lateral sclerosis, multiple sclerosis, Wilson's disease, and a disease group called neurodegeneration with brain iron accumulation (NBIA), which is a clinical syndrome comprised of a genetically heterogeneous group of disorders. NBIA is characterized by iron accumulation in different regions of the brain, mainly within the basal ganglia, with the symptoms of movement disorders (dystonia–parkinsonism, cerebellar ataxia), pyramidal dysfunction, dysgnosia, neuropsychiatric abnormalities, and early death. The diagnosis of NBIA relies upon iron accumulation detected by brain MRI combined with clinical features and is finally confirmed by gene analyses. It includes but is not limited to Friedreich's ataxia, aceruloplasminemia, neuroferritinopathy, and pantothenate kinase‐associated neurodegeneration. The genetic basis of the majority of NBIA disorders has been elucidated so further detail is not discussed here.^217^

## 4 FERROPTOSIS: THE ENDING OF IRON OVERLOAD

Ferroptosis is a newly identified iron‐dependent form of regulated cell death, morphologically characterized by a normal nucleus, but shrinking mitochondria with condensed membrane density, a reduction of mitochondrial cristae, and ruptured outer membrane compared with programmed cell death such as apoptosis and autophagy.^218^ In recent years, the mechanism of the iron homeostasis imbalance represented by ferroptosis in the progression of neurodegenerative diseases has been supported by increasing amounts of evidence. Ferroptosis, characterized by altered brain iron homeostasis, dysregulation of the antioxidant system, and oxidative damage, plays an important role in the progression of neurodegenerative diseases such as stroke, AD, PD, and so on.^116^, ^219^, ^220^, ^221^, ^222^, ^223^, ^224^, ^225^, ^226^ Therefore, elucidating the mechanisms involved in ferroptosis is crucial for the treatment of neurodegeneration. Taking the classical mechanism of ferroptosis as an example, starting from the accumulation of intracellular iron, ferroptosis follows closely behind the trilogy: (i) mass production of ROS; (ii) massive peroxidation of phospholipids (PLs) containing polyunsaturated fatty acids (PUFA‐PL) in the plasma membrane; (iii) collapse of the antioxidant system caused by reduced GSH (Figure 1).

### 4.1 Mass generation of ROS

ROS generation is an important prerequisite for lipid peroxidation and ferroptosis. Under physiological conditions, intracellular ROS mainly comes from the respiratory chain in mitochondria, where the redox reaction is approaching equilibrium. Generally, incompletely reduced forms of dioxygen, such as superoxide anion (O_2_^•−^) and H_2_O_2_, are produced at considerably high rates (about 1−4% dioxygen undergoes a reduction reaction) in the process of reducing dioxygen to water completely. And such reduced dioxygen in vivo can be generated from other enzymes, such as oxygenases, oxidases, and peroxidases. For example, H_2_O_2_ produced by xanthine oxidase is able to significantly aggravate ischemic/reperfusion injury.^227^, ^228^ Reduced dioxygen can not only cause or promote oxidative stress directly, but also lead to the mass generation of secondary products with oxidative activity and destructiveness by reacting with other specific substances, such as hydroxyl radical (OH^•^) which is extremely toxic and produced by the Fenton reaction.

$$Fe^{2+}+H_{2}O_{2}\to Fe^{3+}+OH^{-}+OH^{•}$$

OH^•^ is able to react with multifarious biological molecules, such as DNA, proteins, and unsaturated lipids, thus damaging cell functions.^229^

### 4.2 Massive peroxidation of PUFA‐PL

Peroxidation of PUFA‐PL is the key to executing ferroptosis. When peroxidation exceeds the buffer capacity provided by the antioxidant system, it will lead to the fatal accumulation of lipid peroxides on the cell membrane and the subsequent collapse and rupture of the cell membrane, then ferroptosis happens as a result. Acyl‐CoA synthetase long‐chain family member 4 (ACSL4) and lysophosphatidylcholine acyltransferase 3 (LPCAT3) are acknowledged as the key regulatory factors for PUFA‐PL synthesis. ACSL4 catalyzes the addition of CoA to the long‐chain polyunsaturated bonds of PUFA, thereby promoting the formation of coenzyme A‐activated polyunsaturated fatty acid (PUFA‐CoA), followed by the esterification of PUFA‐CoA to PLs mediated by LPCAT3.^230^, ^231^ Lipid peroxidation is initiated in two ways: enzymatic reaction meditated by lipoxygenases (LOXs) and nonenzymatic reaction, which is iron dependent. The formation of lipid radicals (R‐OOH) can be directly catalyzed by LOXs, a family of enzymes that contains nonheme iron for their activity and is critical for multiple signaling pathways, such as arachidonic acid LOXs.^232^ Multiple enzymes have been proven to drive lipid peroxidation, for instance, 15‐LOX, by binding to phosphatidylethanolamine binding protein 1, is able to switch the substrate specificity of the enzyme from free PUFAs to PUFA tails of PLs thus promoting the peroxidation of PUFA‐PL, 12‐LOX is crucial to ferroptosis mediated by p53, and cytochrome P450 oxidoreductase is involved in lipid peroxidation in ferroptosis.^233^, ^234^, ^235^ Moreover, these enzymes are all iron dependent.

**FIGURE 3:**
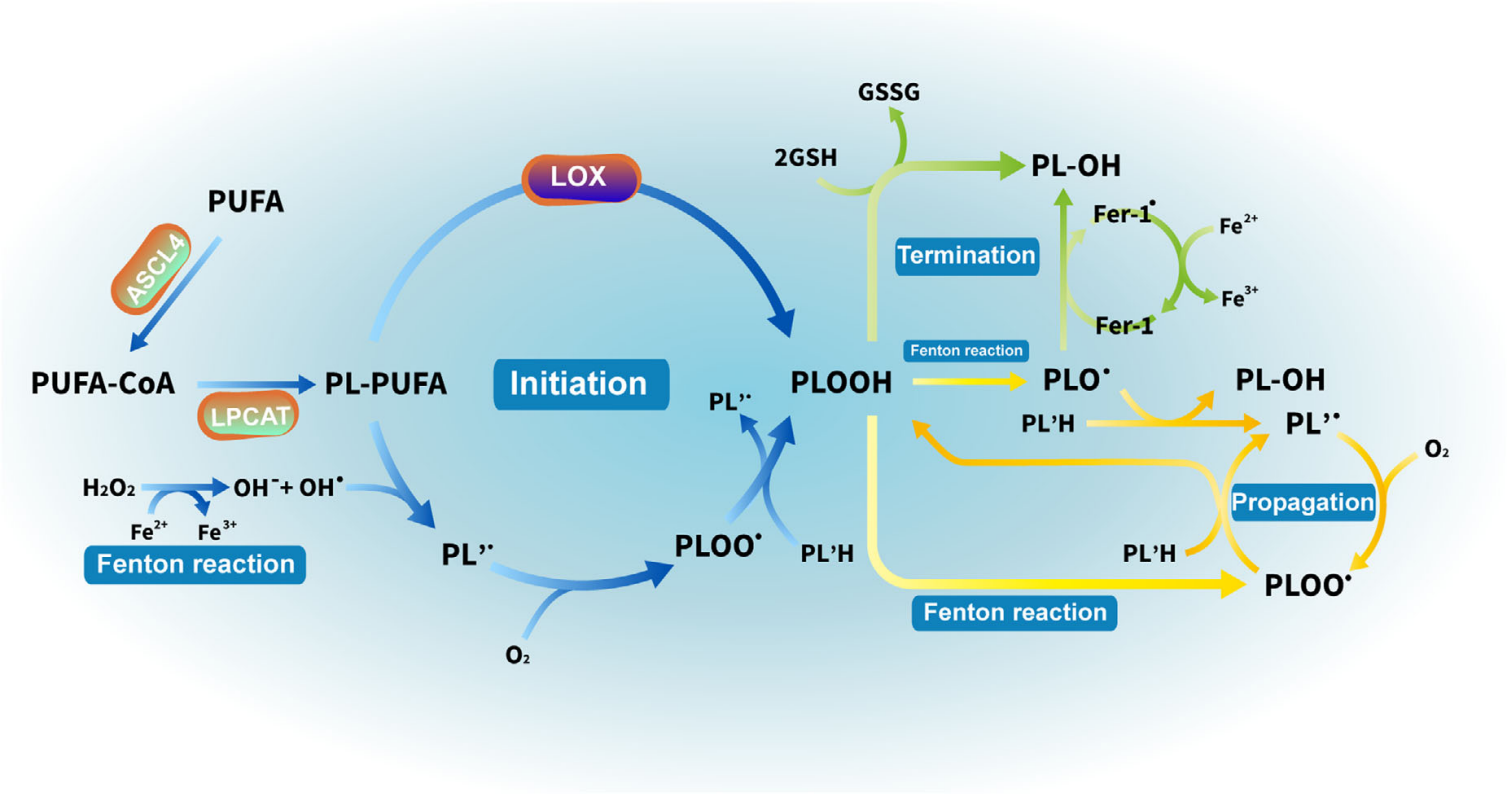
The three phases of lipid peroxidation. (i) Initiation is the process that generates radical compounds (PLOOH/PLOO•) from nonradical molecules (PL‐PUFA). (ii) Propagation, starting from lipid peroxides (PLOOH), is a peroxidative chain reaction giving rise to new radicals while the number of radicals remains constant. (iii) Termination is the process in which the peroxidative chain is broken by donating electrons to radical compounds so that transferring the active product (radical compounds) to a stable one (PL‐OH). It should be noted that the iron‐involved Fenton reaction, which participates in the process of initiation and propagation, is a free electron provider. PUFA, polyunsaturated fatty; ACSL4, acyl‐CoA synthetase long‐chain family member 4; PUFA‐CoA, coenzyme A‐activated polyunsaturated fatty acid; LPCAT3, lysophosphatidylcholine acyltransferase 3; PL‐PUFA/PL/PL'H, phospholipids containing polyunsaturated fatty acids; LOX, lipoxygenase; PLOO•, phospholipid peroxy radical; PLOOH, phospholipid hydroperoxide; PLO•, the phospholipid alkoxyl radical; GSH, glutathione; GSSG, oxidized glutathione; Fer‐1, ferrostatin‐1; PL‐OH, phospholipid hydroxides.

Lipid peroxidation occurs mainly through nonenzymatic pathways, which, as ROS dependent, can be divided into three phases: initiation, propagation, and termination (Figure 3). In the initiation phase, labile oxidants (OH^•^) grab hydrogen atoms from the bisallylic methylene of a membrane PL‐PUFA, leaving a phospholipid radical (PL’^•^) centered on carbon. The PL’^•^ cannot keep self‐stable and reacts with oxygen producing another substrate with oxidative capacity—phospholipid peroxy radical (PLOO^•^), which is able to extract hydrogen atoms from surrounding PL to generate a phospholipid hydroperoxide (PLOOH) along with a new PL’^•^. PLOOH is an essential free radical precursor that initiates the next peroxidation chain cycle(progression) in which the Fenton reaction is involved. As a circulating substrate in the phospholipid peroxidation, if the generation of PLOOH in neurons exceeds the capacity of the peroxide scavenging system, it can propagate peroxidation to adjacent PUFA‐PLs on the PL membrane in the presence of labile iron, thereby disrupting the permeability and fluidity of the membrane and ultimately causing ferroptosis.^236^ Antioxidants, like GSH, can terminate radical propagation by donating electrons to radical compounds without being a radical, which is a primary mechanism of defense against lipid peroxidation and other oxidative damage.

### 4.3 Reduction of GSH: the collapse of antioxidant system

As the major intercellular antioxidant against oxidative stress, GSH plays a critical role in protecting cells from ROS attack.^237^ By binding to intracellular Fe^2+^, GSH is able to effectively reduce the labile iron pool, which in turn reduces the generation of lipid peroxides and ROS mediated by Fe^2+^. The import of raw materials for GSH synthesis is dependent on the system xc^−^ and the excitatory amino acid transporter (EAAT). The system xc^−^ on the cell membrane, as a heterodimeric protein complex, is composed of solute carrier family 3 member 2 (SLC3A2) and solute carrier family 7 member 11 (SLC7A11) and serves its function by importing the extracellular cystine along with exporting the intracellular glutamate by a ratio of 1:1.^238^ The cystine transferred into the cell is decomposed into cysteine for further reaction. Neuronal glutamate transporters, mainly EAAT3 in postsynaptic neurons, exert functions by clearing extracellular excess glutamate to maintain a normal concentration gradient of intra‐to‐extra cellular glutamate for normal functioning of the system xc^−^. In addition, EAAT3 also functions as a unidirectional cysteine import channel that facilitates cysteine entry into the cell.^239^ Under the catalysis of γ‐glutamylcysteine synthetase, cysteine combines with glutamate to form γ‐glutamylcysteine, which will next combine with glycine to form GSH.^240^, ^241^

Under physiological conditions, most glutamates are rapidly cleared by EAAT3 at the postsynaptic membrane after exerting its physiological role at the neuronal synapse, leaving extracellular glutamate at a low level, which is critical to the normal function of synapses and neural circuits.^242^ However, pathological conditions, such as ischemia after stroke, cause the dysfunction of EAAT3, resulting in excess accumulation of extracellular glutamate, which inhibits the normal function of the system xc^−^.^246^ This is because the glutamate/cystine exchange proceeding depends on the gradient of glutamate toward the cell, rather than consuming adenosine triphosphate actively. With the excess accumulation of extracellular glutamate, cystine turnover is inhibited, ultimately leading to a rapid decline in GSH synthesis. Therefore, normal GSH content is essential for the maintenance of the cellular antioxidant system. The lower baseline plasma GSH levels were reported to be associated with an increased risk of developing neurodegenerative diseases, such as AD and PD.^244^, ^245^

In addition, the body's antioxidant system also faces the loss of enzyme activity in GSH synthesis, which is crucial to the antioxidant response. As an important antioxidant enzyme, GPX4 conducts its biological activity by converting two molecules of GSH to oxidized glutathione (GSSG) along with reducing phospholipid hydroperoxides (PL‐OOH) to phospholipid hydroxides (PL‐OH), to prevent the accumulation of lipid peroxides, which is toxic.^246^ The reserve of GSH is consumed by constant Fenton reaction induced by mass labile iron during stroke and neurodegenerative diseases, leading to a decline in the concentration of GSH as enzymatic reactants, which in turn inhibits GPX4 activity.^247^ Moreover, evidence shows that promoting GPX4 activity and supplementation of GSH in neurodegenerative diseases and stroke both can mitigate oxidative stress and inflammatory pathology related to ferroptosis, thereby contributing to better preservation of executive functioning longitudinally.^244^, ^248^, ^249^, ^250^, ^251^, ^252^

Therefore, GSH supplementation appears to be a promising target for stroke and neurodegenerative diseases. However, due to the isolation of BBB and the omnipresent γ‐glutamyl transpeptidase, which is responsible for GSH cleavage, GSH supplementation in vitro fails to achieve the ideal effect in neurological protection.^253^, ^254^ It was reported in recent years that some stable GSH analogs, prodrugs, and GSH‐coupled nanocarriers, such as ψ‐GSH, l‐cysteine, and γ‐glutamylcysteine, can overcome the disadvantages of GSH administration to achieve the aim of elevating GSH levels in the brain.^249^, ^255^, ^256^, ^257^, ^258^, ^259^ Therefore, drug development in GSH remains to be explored and will focus on the delivery and functional properties of drugs. In addition, although GPX4 plays the central part in ferroptosis inhibition, researchers have found four other systems capable of suppressing ferroptosis act independently of GPX4 in the past few years, including ferroptosis suppressor protein 1 (FSP1)/CoQ10 pathway, GTP cyclohydrolase 1 (GCH1)/tetrahydrobiopterin (BH4) pathway, dihydroorotate dehydrogenase (DHODH) /CoQ Pathway, and p62/Keap1/Nrf2 Pathway.^260^, ^261^, ^262^, ^263^, ^264^, ^265^ Understanding new mechanisms will contribute to developing new targeted drugs and clinical trials.

## 5 THERAPEUTIC AGENTS FOR TREATING IRON ACCUMULATION AND FERROPTOSIS: IRON CHELATORS AND FERROPTOSIS INHIBITORS

The accumulation of labile iron is an initiating trigger of ferroptosis, which is followed by the peroxidation of membrane lipids by the mass production of ROS, ultimately leading to neuronal death and impairment of neurological function. Therefore, keeping labile iron content in control or inhibiting the occurrence of ferroptosis both work for neurological protection.

### 5.1 Iron chelators

Deferoxamine (DFO), the most widely used iron chelator approved by the Food and Drug Administration in America, has been used clinically for a long time as a first‐line therapeutic agent to scavenge excess iron in diseases such as thalassemia, and its effectiveness as a neuroprotectant has been demonstrated in experimental stroke models.^266^, ^267^ The rationale that iron chelators exert neuroprotective effects is mainly based on chelating and scavenging free iron in the blood and enhancing iron excretion by the urine. When Fe^3+^ comes into contact with DFO, the linear chains of DFO perssad will wrap around Fe3^+^, forming a stable complex containing iron, blocking the entry of Fe^3+^ into the Haber Weiss reaction and interrupt the toxic redox cycle, ultimately reducing the generation of ROS and the accumulation of lipid peroxides.^268^ The iron chelators reported in recent years and their related mechanisms are summarized in Table 1.

**TABLE 1:** Iron chelators for neurological diseases

Name	Mechanism/effect	Target disease	References
Deferiprone	↓Iron content of dentate, caudate nucleus, and substantia nigra ↑Motor ability ↑Memory ability and reduce the burden of amyloid and p‐tau ↑The mean lifespan without anemia in ALS mice models Stabilize disease progression of NBIA	PD, AD, ALS, NBIA	^269^, ^270^, ^271^, ^272^, ^273^, ^274^, ^275^

Deferasirox	↓The intracellular Fe^2+^ accumulation, ROS, and lipid peroxidation Maintain BBB permeability ↓Neuroinflammation	HS, IS	^266^, ^276^
TPP‐DFO	High affinity for mitochondrial iron Antioxidant activity	NBIA	^277^

Quercetin	Chelate iron ions inside and outside the cell Shuttle intracellular free iron ions to extracellular space through GLUTs (when quercetin's concentrations≦ 1 μM) ↑Hepcidin expression by Nrf2 upregulation	Stroke, AD, PD	^278^, ^279^, ^280^
M30	↑ HIF‐1 protein, growth factors, and antioxidant enzymes	AD, PD, ALS	^281^

Curcumin	Iron chelation ↓Aβ	AD	^282^
Kaempferol	↑ Nrf2/SLC7A11/GPX4 signaling pathway	IS	^283^

PBPT	Iron chelation ↓Copper‐mediated amyloid‐β aggregation ↓Oxidative stress ↓AChE activity ↑Autophagy	AD	^284^
Naringin	Iron chelation ↓ Aβ	AD	^285^

NBMI	Iron chelation preferential to labile Fe^2+^	–	^286^
YL‐939	↑Ferritin ↑NCOA4 and ferritinophagy	–	^287^

*Note*: ↑, activate/upregulate; ↓, inhibit/suppress.

Abbreviations: PD, Parkinson's disease; AD, Alzheimer's disease; ALS, amyotrophic lateral sclerosis; NBIA, neurodegeneration with brain iron accumulation; ROS, reactive oxygen species; BBB, blood–brain barrier; HS, hemorrhagic stroke; IS, ischemic stroke; TPP, triphenylphosphonium; Aβ, β‐amyloid; Nrf2, nuclear factor erythroid 2‐related factor 2; HIF‐1, hypoxia‐inducible factor‐1; SLC7A11, solute carrier family 7 member 11; GPX4, glutathione peroxidase 4; AchE, acetylcholinesterase; NBMI, N, N’‐bis(2‐mercaptoethyl)isophthalamide; NCOA4, nuclear receptor coactivator 4.

Interestingly, it was reported that two new forms of DFO (PEGylated DFO and liposome‐DFO) seem to achieve results at opposite poles in the treatment of stroke, with the former a greater half‐life, lower cytotoxicity, and better iron chelation than traditional DFO, while the latter aggravating nerve damage and delaying neurological function recovery, which suggests that iron chelators might be a promising therapy for treating iron‐overload conditions in the clinic but still need to be further verified by lots of experiments.^94^, ^288^ In addition, though the safety of DFO has also been further validated in clinical trials, the starting and during time, and dosage of iron chelators should be individualized and disease‐specific, which are worthy of further exploration.^289^ And as we have discussed above, the irrational use of iron chelators may be counterproductive.

### 5.2 Ferroptosis inhibitors

In contrast, ferroptosis inhibitors mainly function to break the autoxidation of chain‐propagating ROS and prevent excess ROS from lipid peroxidation. Ferrostatin‐1 (Fer‐1) has been regarded as the most potential ferroptosis inhibitor and a standard drug in experimental studies to judge the effects of other inhibitors.^218^, ^290^ As a member of the lipophilic radical‐trapping antioxidant (RTA) family, the mechanism of Fer‐1 in inhibiting ferroptosis has been widely explored in recent years. The latest researches suggest that Fer‐1 can bind the 15‐LOX/PEBP1 complex and disrupt its allosteric movement required for catalysis, thus inhibiting the catalytic activity of 15‐LOX for lipid peroxidation and further blocking ferroptosis caused by GPX4 insufficiency.^218^, ^291^ And Fer‐1 is capable to alleviate angiotensin II‐induced inflammation and ferroptosis by suppressing the ROS levels and activating the Nrf2/HO‐1 signaling pathway.^292^, ^293^ Moreover, Fer‐1 is associated with the downregulated expression of ferroptosis‐related genes and products (ireb2 and PTGS2), leading to the clearance of iron and ROS.^294^ In addition, Liprostatin, another member of the RTAs, has similar mechanisms to Fer‐1 while better absorption, distribution, and effects with lower doses in the body.^295^ Moreover, several other enzymes, like ACSL4, LPCAT3, and LOX, can promote lipid peroxidation during ferroptosis, therefore, drugs that inhibit these enzymes antagonize the onset of ferroptosis. The ferroptosis inhibitors reported in recent years and their related mechanisms are summarized in Table 2.

**TABLE 2:** Ferroptosis inhibitors for neurological diseases

Name	Mechanism/effect	Target disease	References
N‐acetylcysteine	↓Toxic arachidonic acid products of nuclear ALOX5	HS	^296^

PD146176	↓SSAT1/ALOX15 axis	IS	^297^
Naotaifang	↓TfR1 and DMT1 ↑SLC7A11, GPX4 and GSH	IS	^298^

ADA‐409‐052	↓TBHP‐induced lipid peroxidation ↓Activation of BV2 microglia Maintain mitochondrial morphology	IS	^299^
Thiazolidinediones	↓ACSL4 activity selectively	IS	^300^

Rosiglitazone	↓ACSL4 activity selectively	IS	^301^
(R)‐HTS‐3	↓LPCAT3 activity selectively	–	^302^

Baicalin	↑The mRNA expression of GPX4 and SLC7A11 in the peri‐hematoma brain tissues	HS	^303^
Dauricine	↑GPX4 and glutathione reductase coexpression	HS	^304^

Melatonin	↓5‐LOX level by circPtpn14/miR‐351‐5p signaling	TBI	^305^
DHI	↑SATB1/SLC7A11/HO‐1 pathway	IS	^306^

β‐Caryophyllene	↑Nrf2/HO‐1 signaling pathway	IS	^307^
Eriodictyol	Aβ aggregation and p‐tau ↑Nrf2/HO‐1 pathway through VDR	AD	^308^

α‐Lipoic acid	↓p‐tau Iron redistribution mediated by TfR and Fpn1	PD	^309^
TS, TP	Inhibit 15‐LOX by competing for the substrate binding site of PUFA	_	^310^

*Note*: ↑, activate/upregulate;↓, inhibit/suppress.

Abbreviations: HS, hemorrhagic stroke; SSAT1, spermidine/spermine N1‐acetyltransferase 1; ALOX5, arachidonate 5‐lipoxygenase; IS, ischemic stroke; TfR1, transferrin receptor 1; DMT1, divalent metal transporter 1; SLC7A11, solute carrier family 7 member 11; GPX4, glutathione peroxidase 4; GSH, glutathione; TBHP, tert‐Butyl hydroperoxide; ACSL4, Acyl‐CoA synthetase long‐chain family member 4; LPCAT3, lysophosphatidylcholine acyltransferase 3; 5‐LOX, 5‐lipoxygenase; TBI, traumatic brain injury; DHI, danhong injection; SATB1, special AT‐rich sequence‐binding protein 1; SLC7A11, solute carrier family 7 member 11; HO‐1, heme oxygenase‐1; Nrf2, nuclear factor erythroid 2‐related factor 2; Aβ, β‐amyloid; VDR, vitamin D receptor; p‐tau, phosphorylated tau; TfR, transferrin receptor; Fpn1, ferroportin 1; TS, α‐Tocopherol succinate; TP, α‐Tocopherol phosphate; 15‐LOX, 15‐lipoxygenase; PUFA, polyunsaturated fatty acids.

Apart from pharmacological inhibitors, there are two essential fat‐soluble vitamins (vitamin E and vitamin K) in the human body that can act as antioxidant molecules and exert antiferroptosis effects. Vitamin E terminates the propagation reactions by providing hydrogen atoms to PLOO∙ along with the formation of vitamin E free radicals (TOC∙). Finally, nonradical products are generated from the reaction of TOC∙ with another PLOO∙, representing an interruption of the oxidative transport chain.^311^ Vitamin E can also regulate ferroptosis by LOX inhibition.^312^ Besides, vitamin K was shown to confer robust antiferroptotic activity via its reduced form (VKH_2_), which is a potent RTA and PL peroxidation inhibitor.^313^ Ferroptosis suppressor protein 1 (FSP1), a NAD(P)H‐ubiquinone reductase, plays a crucial role in reducing vitamin K to VKH_2_. Given the inhibitory role of vitamin E and vitamin K in ferroptosis, there may be nutritional approaches to the treatment of poststroke and neurodegenerative diseases combined with ferroptotic inhibitors

## 6 CONCLUSION AND PROSPECTS

**FIGURE 4:**
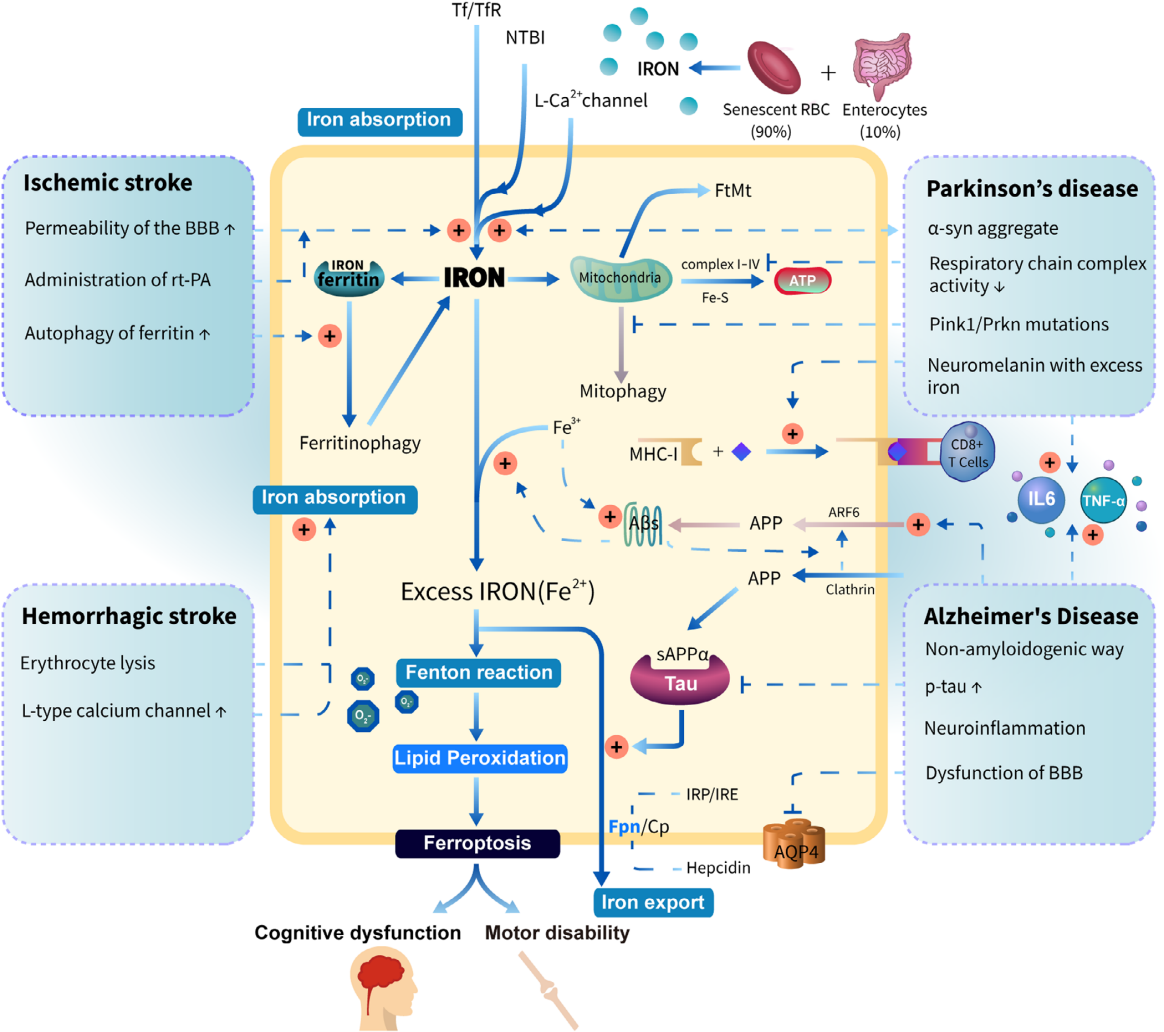
A generalization of the pathogenesis of brain iron accumulation in ischemic stroke, hemorrhagic stroke, Alzheimer's disease, and Parkinson's disease. Stroke greatly increases the import of brain iron, resulting in iron accumulation. In contrast, the progression of neurodegeneration and iron accumulation are mutually reinforcing. Mass labile iron, as a central link in stroke and neurodegenerative diseases, involves in the Fenton reaction and triggers the imbalance between the oxidation reaction and the antioxidant system in the neurological system, which leads to ferroptosis and then poor outcomes for patients. Tf, transferrin; TfR, transferrin receptor; NTBI, nontransferrin‐bound iron; Fpn, ferroportin; Cp, ceruloplasmin; FtMt, mitochondrial ferritin; MHC‐I, major histocompatibility complex class I; APP, amyloid precursor protein; ARF6, ADP ribosylation factor 6; p‐tau, phosphorylated tau; BBB, the blood–brain barrier; AQP4, aquaporin‐4. Note: "+ in the red cycle” means to promote.

Iron is an important micronutrient for life maintenance, and it is closely related to the physiological metabolism of the human body. The pathogenesis of iron metabolism imbalance in brain diseases has been the focus of researchers in recent years. The regulation of iron homeostasis in the brain is very strict and involves the blood–brain barrier, the IRP/IRE system, the regulation of hepcidin, and the expression of many transporter proteins and enzymes. However, acute onset represented by stroke and chronic neurodegeneration represented by AD and PD interfere with the maintenance of brain iron homeostasis through their unique pathological mechanisms, leading to excessive accumulation of iron in the brain (Figure 4), which is evidenced by neuroimaging, pathology, and animal experiments. Excessive accumulation of iron in the brain triggers ferroptosis, a specific pattern of apoptosis, which significantly affects the prognosis of patients. Since its discovery and identification in 2012, many advances have been made to understand the mechanisms of ferroptosis. Many pharmacological agents like iron chelators and ferroptosis inhibitors have been used to bring iron metabolism imbalance back on track and modulate the ferroptotic response to attenuate morbidity and mortality. In contrast, many unresolved problems in iron metabolism and ferroptosis still need to be tackled in the future.

In terms of pathological mechanisms, although the link between brain iron accumulation and neurodegeneration is increasingly being demonstrated, it is not clear yet whether iron deposition plays a cause or a consequence of these diseases. In addition, whether iron accumulation in the early stages of the disease is an initial reaction for self‐protection of the nervous system in facing noxious stimuli remains unclear, if so, then what are the thresholds for iron accumulation to be neuroprotective or ferroptosis‐inductive? On top of these, we still face a conundrum: who is the executor of ferroptosis? In terms of clinical treatments, although iron chelators have been proven beneficial in diseases related to iron metabolism imbalance, the dose and timing pattern of their administration in stroke patients remains to be determined. And the efficacy of clinical trials of ferroptosis inhibitors is still unknown. Developing key molecular markers to identify disease progression in order to facilitate the rational use of clinical drugs will be valuable. At last, the effects of comorbidities on iron metabolism and iron chelation must be taken into consideration in the future. Integrating the pathogenetic background of patients with iron chelation therapy to achieve the purpose of personalized and precise treatment in the future is worth looking forward to.

In summary, our understanding of the mechanisms involved in the regulation of iron homeostasis and ferroptosis has advanced enormously in the past several decades. Elucidating the mechanisms by which iron accumulation and ferroptosis occur not only helps to gain insight into the process of iron homeostasis imbalance and neurodegeneration but also facilitates the discovery of new therapeutic targets for related brain diseases. Nevertheless, many unknown aspects of iron metabolism and ferroptosis in brain disease remain to be unfolded, which requires a huge effort in basic animal research and clinical drug research.

## AUTHOR CONTRIBUTION

Z. J. G. proposed the topic; Z. J. G., W. L. M., and Z. W. S. analyzed the structure and intellectual content of this review and made critical revisions. L. H. N. prepared, revised the manuscript, and drew the figures. All the authors agreed to publish this review.

## CONFLICT OF INTEREST STATEMENT

The authors declare no conflict of interest.

## ETHICS STATEMENT

Not applicable.

## Data Availability

Not applicable.
